# Neurotoxicity of PBDEs: Dingemans et al. Respond

**DOI:** 10.1289/ehp.1103907R

**Published:** 2011-08-01

**Authors:** Milou M.L. Dingemans, Martin van den Berg, Remco H.S. Westerink

**Affiliations:** Institute for Risk Assessment Sciences Utrecht University, Utrecht, the Netherlands, E-mail: m.dingemans@uu.nl

Banasik and Suchecka express their discontent with our recent review on the (in-)direct neurotoxic effects of parent and hydroxylated (OH-) polybrominated diphenyl ethers (PBDEs) on the (developing) nervous system (Dingemans et al. 2011). Their main discontent appears to be once more related to the experimental design in a number of cited behavioral studies. However, our aim was to identify and review the mechanisms underlying the observed adverse (behavioral) effects, not to evaluate the experimental design of behavioral studies within a regulatory setting. Nonetheless, approximately 10% of our review was dedicated to describing a number of behavioral studies [12 different studies from seven different research groups, including a 2008 EPA study (Gee and Moser 2008)] that all indicated the occurrence of neurobehavioral effects following developmental exposure to PBDEs. We used this information to create a starting point for the main part of our review of direct and indirect cellular and molecular mechanisms. For readability and space limitations, we were not able to include all studies, concerns, or critiques that have ever been raised. The absence of a citation to a particular study does not mean that we regard it as less credible.

The view that (developmental) exposure to PBDEs induces adverse neurotoxic effects is widely supported by numerous *in vivo*, *ex vivo*, and *in vitro* studies reporting both structural and functional effects (Dingemans et al. 2011). For some time, a lively discussion has been taking place within the scientific community on the experimental design for behavioral developmental neurotoxicity studies for regulatory purposes, in particular considering the statistical unit (Alcock et al. 2011). In short, there is disagreement about whether direct dosing of pups precludes the need to control for litter effects (e.g., Eriksson 2008; Hardy and Stedeford 2008). However, we did not address this topic in our paper because we consider the potential occurrence of a litter effect to be irrelevant for the reviewed cellular and molecular *in vitro* studies, which all indicate that exposure to PBDEs induces neurotoxic effects.

Critical remarks can be found throughout our review (Dingemans et al. 2011), but they are related to cellular and molecular findings, data gaps, or aspects that warrant further investigation. Our main conclusions are related to the specific (developmental) neurotoxic hazard of OH-PBDEs compared with that of their parent congeners via direct neurotoxicity and thyroid disruption. We also pointed out the need to further investigate the impact of active metabolites, concentrations of PBDEs and metabolites in the (developing) brain, and the potentially increased neurotoxic hazard following exposure to mixtures of different environmental contaminants.

Nonetheless, Banasik and Suchecka raise an important issue: the existence of differences in experimental designs for *in vivo* investigation of (developmental) neurotoxicity. Differences exist in the selection of investigated end points and also in methodologies for the investigation of a specific end point, as reviewed for effects on motor activity by brominated flame retardants (Williams and DeSesso 2010). These differences in experimental design may underlie observed differences in sensitivity to detect neurotoxicity, possibly because of differences in biokinetics and exposure during sensitive windows of development. Fortunately, much effort is taking place in the scientific community to optimize experimental designs at different levels of biological complexity, including (developmental) neurobehavioral studies. Although a critical review on the impact of different experimental designs for *in vivo* (developmental) neurotoxicity studies would be very useful, it was beyond the scope of our review (Dingemans et al. 2011).
